# A cluster randomized trial on inspiratory effort-targeted pressure support adjustment strategy in patients undergoing assisted mechanical ventilation: protocol for the IT-PSV study

**DOI:** 10.3389/fmed.2024.1483976

**Published:** 2024-11-08

**Authors:** Wen-Yi Lu, Ming-Yue Miao, Ran Gao, Yan-Lin Yang, Linlin Zhang, Li Weng, Feng-Xue Zhu, Lei Liu, Jian-Xin Zhou

**Affiliations:** ^1^Department of Critical Care Medicine, Beijing Tiantan Hospital, Capital Medical University, Beijing, China; ^2^Clinical and Research Center on Acute Lung Injury, Emergency and Critical Care Center, Beijing Shijitan Hospital, Capital Medical University, Beijing, China; ^3^Medical Intensive Care Unit, Peking Union Medical College Hospital, Beijing, China; ^4^Department of Critical Care Medicine, Peking University People's Hospital, Beijing, China; ^5^Department of Scientific Research, Beijing Shijitan Hospital, Capital Medical University, Beijing, China

**Keywords:** pressure support ventilation, muscle pressure index, inspiratory effort, protective ventilation, setting

## Abstract

**Background:**

Pressure support ventilation (PSV) is one of the most frequently used ventilator modes in the intensive care unit (ICU). The successful implementation of PSV depends on matching the patient’s inspiratory effort with the ventilator support. In clinical practice, the pressure support level is usually set and adjusted according to tidal volume and respiratory rate. However, these parameters may not fully represent the patient’s effort. Previous studies have shown that pressure muscle index (PMI), which is measured as the difference between the peak and plateau airway pressure during an end-inspiratory airway occlusion, could reliably determine the low and high inspiratory effort during PSV. Herein we present the study protocol for the Inspiratory effort-Targeted Pressure Support Ventilation (IT-PSV) trial to determine the effect of a PMI-targeted pressure support setting strategy on clinical outcomes in patients undergoing PSV.

**Methods and analysis:**

This is a cluster randomized controlled trial. Sixteen ICUs in academic hospitals will be included, eight of which will be randomly allocated to the PMI-targeted group and eight to the tidal volume/respiratory rate-targeted group. Before the initiation of the study, a four-week comprehensive training program, which includes courses of PSV initiation, pressure support adjustment, and weaning process, will be conducted for all staff in the participating ICUs. Adult patients with acute hypoxic respiratory failure and undergoing PSV within 24 h will be included. Pressure support setting and adjustment will follow the strategy according to the grouping. The primary outcome is the ventilator-free days at 28 days after enrollment. The patients will be followed up until successful weaning or separation of mechanical ventilation, death, hospital discharge, or until 28 days after randomization, whichever comes first.

**Discussion:**

The IT-PSV trial will examine the effect of an inspiratory effort-targeted PSV setting strategy on the duration of mechanical ventilation. If positive, it will provide a new physiological-based PSV management that could potentially facilitate protective assisted ventilation.

**Clinical trial registration:**

ClinicalTrials.gov, identifier NCT06526598.

## 1 Introduction

Mechanical ventilation is a necessary supportive care in the intensive care unit (ICU). Compared to controlled ventilator modes, assisted ventilation, during which the patient’s spontaneous breathing is maintained, can improve gas exchange and lung function, redistribute ventilation and end-expiratory volume to dependent lung areas, and prevent respiratory muscle atrophy (1). An international prospective cohort study demonstrated that the use of assisted ventilation had increased over the past two decades, and pressure support ventilation (PSV) had become the most frequently used assisted mode (2). However, preserving spontaneous breathing during positive pressure ventilation is not without safety concerns (3, 4). Under mechanical ventilation, against positive airway and alveolar pressure, vigorous inspiratory effort, especially in patients with acute lung injury, may dramatically increase global and regional lung stress and strain (3, 4). This is considered the primary mechanism of the patient’s self-inflicted lung injury (5). On the contrary, excessive support and/or deep sedation may result in low effort, which may lead to disuse atrophy of the inspiratory muscles, thereby prolonging the duration of mechanical ventilation (6). Therefore, monitoring and controlling inspiratory effort during assisted ventilation is of important clinical significance.

Although initially designed as a ventilator mode for the weaning process, PSV is widely used to assist ventilation during the acute phase of critical illness (2, 7). During PSV, the ventilator provides a constant level of pressure for breaths, which are all triggered by the patient (8). The successful implementation of PSV depends on matching the patient’s inspiratory effort with the ventilator support (7). Although esophageal pressure is used as a reference standard for inspiratory effort assessment (9, 10), its routine application is limited by the requirement of special equipment and complex computation processes (11, 12). In clinical practice, pressure support levels are usually set and adjusted according to tidal volume (VT) and respiratory rate (RR); however, these parameters may not fully represent the patient’s effort (7). Previous studies have also shown that over-assistance under PSV is not uncommon based on this setting strategy (13, 14). An easily accessible instrument is required to target the support level with the patient’s inspiratory effort at the bedside.

During PSV, an end-inspiratory airway occlusion can induce a plateau airway pressure (Paw). The difference between the peak and plateau Paw was named as pressure muscle index (PMI) (15). Studies have shown that PMI is closely correlated with inspiratory effort determined by esophageal pressure-derived indices (15–17) and electromyography of respiratory muscles (18). Our previous study also found that PMI could reliably discriminate low and high inspiratory effort with respective cutoff values of 0 and 2 cmH_2_O (19), and these cutoff values could be reliably used as a parameter to predict low and high contribution of patient’s effort during PSV (20). These results suggest the potential use of PMI as an effort-targeted indicator for PSV setting and adjustment. Moreover, PMI can be directly measured on the ventilator screen without additional equipment, suggesting its convenient bedside use.

With a cluster randomized controlled trial (RCT) design, the Inspiratory effort-Targeted Pressure Support Ventilation (IT-PSV) trial will determine the effect of a PMI-targeted pressure support setting strategy on clinical outcomes in patients undergoing PSV. We are proposing a physiological-oriented assisted ventilation management that, if found effective, could potentially change the clinical practice of mechanical ventilation.

## 2 Methods and analysis

### 2.1 Study design, settings, and ethics

**Figure 1:**
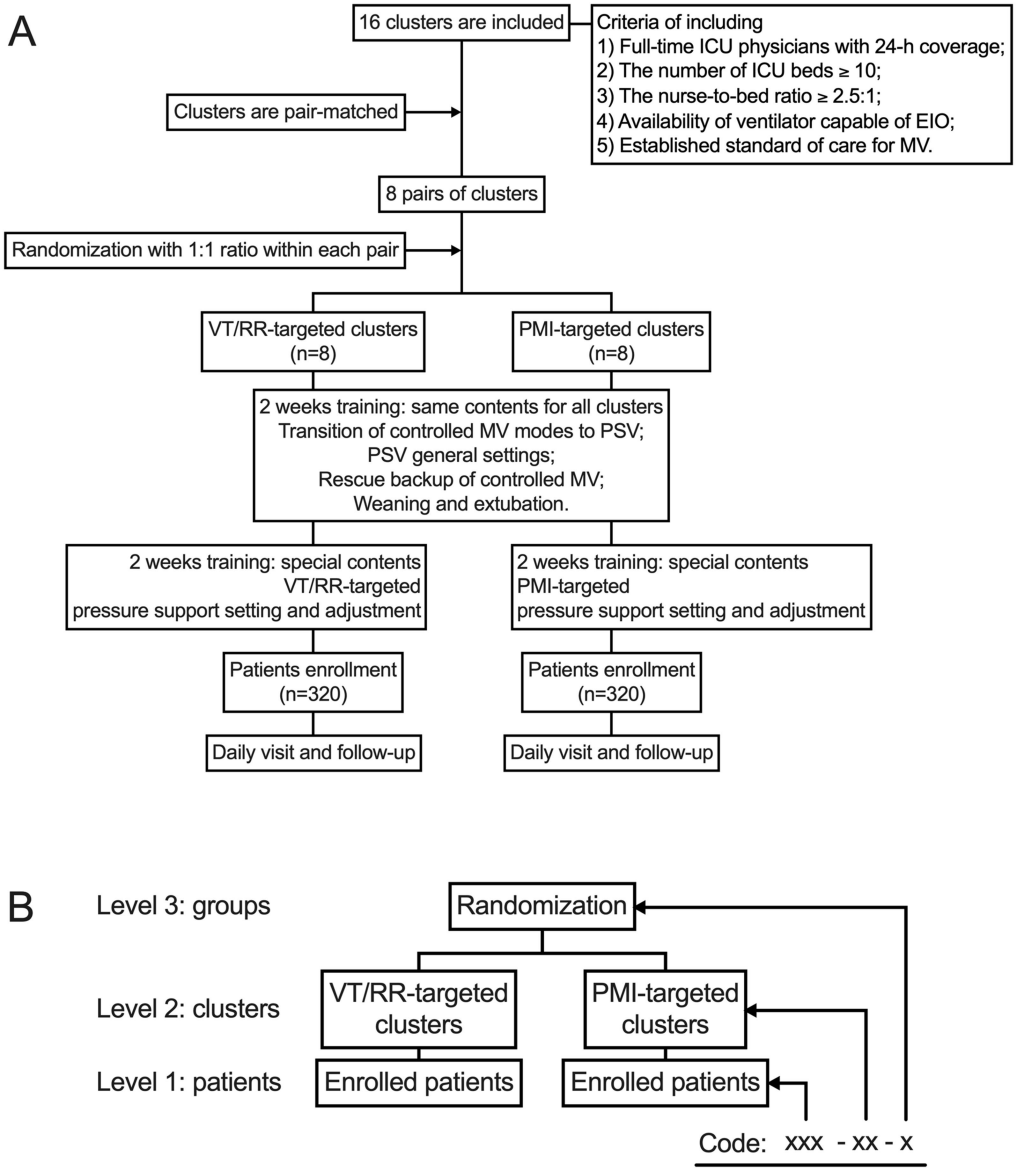
Flow chart of the trial **(A)** and data structure **(B)**. EIO, end-inspiratory occlusion; MV, mechanical ventilation; PMI, pressure muscle index; PSV, pressure support ventilation; RR, respiratory rate; VT, tidal volume.

IT-PSV is a cluster RCT that will test the influence of a PMI-targeted pressure support adjustment strategy on clinical outcomes in patients undergoing PSV. The flowchart of the trial is shown in Figure 1A.

The study protocol has been approved by the Institutional Ethics Committee in Beijing Shijitan Hospital (IIT2024-029-002) and registered at ClinicalTrials.org (NCT06526598). The study design adheres to the standard protocol items for randomized trials (Supplementary material S1 shows the Standard Protocol Items: Recommendations for Interventional Trials [SPIRIT] 2013 Checklist) (21).

Before the start of the study, a central coordination group is formed of three chief investigators (FXZ, LW, and JXZ) and clinical research coordinators. An invitation letter is sent to the directors of ICUs in academic hospitals in Beijing, Tianjin, and Hebei (Jing-Jin-Ji area) in China. The included ICUs have to fulfill the following criteria:

1. The unit is run by full-time ICU physicians with 24 h coverage;
2. The number of beds is more than 10;
3. The nurse-to-bed ratio is more than 2.5:1;
4. Ventilators capable of end-inspiratory airway occlusion are available;
5. Standard clinical practice for mechanical ventilation has been established.

Finally, 16 ICUs (clusters) are included, eight allocated to the PMI-targeted group and eight to the VT/RR-targeted group. A local primary investigator is confirmed in each participating ICU, who will provide structural and scientific leadership and guarantee the integrity and timeliness of data collection. Ethical approval is obtained in each participating hospital.

### 2.2 Randomization

The randomization will be performed by a statistician who will not be involved in the study procedures. To improve power and balance, the 16 participating ICUs (clusters) are matched in pairs with a similar number of beds and volume of patients undergoing mechanical ventilation (22). The ICUs are randomized within the pairs with a 1:1 ratio to either the PMI-targeted (*n* = 8) or VT/RR-targeted (*n* = 8) group.

Due to the nature of the intervention and control, it is not possible to blind participating ICUs, personnel providing patient care in the ICU, and researchers performing data collection. However, the researchers who conduct the statistical analyses will be blinded to the grouping.

### 2.3 Patients

Mechanically ventilated patients admitted to the ICU with acute hypoxic respiratory failure (AHRF) (23) will be consecutively screened daily at 08:00–10:00 morning rounds. Patients undergoing PSV are eligible for inclusion.

#### 2.3.1 Inclusion criteria

1. PSV initiated during the last 24 h, whether transition from controlled modes or primary initiation;
2. Mechanical ventilation expected to be required for at least 24–48 h by responsible physicians;
3. The partial pressure of oxygen in arterial blood (PaO_2_)/inspired oxygen fraction (FiO_2_) ≤ 300 mmHg (measuring at clinical FiO_2_ and positive end-expiratory pressure [PEEP]);
4. No sedation or stable sedation with Richmond Agitation Sedation Scale (RASS) of −2 to +1 or Riker’s Sedation-Agitation Scale (SAS) of 3 to 4 (24).

#### 2.3.2 Exclusion criteria

1. Age younger than 18 years old;
2. Initiation of PSV before ICU admission;
3. Duration of mechanical ventilation longer than 7 days before enrollment;
4. History of neuromuscular diseases;
5. Clinical suspicion of increased intracranial pressure;
6. Presentation with pneumothorax and/or bronchopleural fistula;
7. Extracorporeal support;
8. Moribund conditions;
9. Refusal by the ICU physicians or the patient.

The patients will be enrolled only once during the same hospitalization. Written informed consent will be obtained from the patient or their legal representative.

### 2.4 Study procedures

In both PMI-targeted and VT/RR-targeted groups, standard clinical care for mechanical ventilation is followed according to local routine practice, which is formatted based on international clinical guidelines, except for pressure support adjustment during PSV.

#### 2.4.1 General standard of care for mechanical ventilation

Analgesia is routinely used in mechanically ventilated patients with continuous infusion of fentanyl or remifentanil. Sedation with midazolam, propofol, or dexmedetomidine is used when the patient exhibits agitation and a light sedation level is maintained on RASS of −2 to +1 or Riker’s SAS of 3 to 4 (24).

In patients with AHRF, mechanical ventilation is usually initiated on protective control ventilation, such as volume or pressure control mode, with VT 6–8 mL/kg predicted body weight (PBW), RR to control arterial partial pressure of carbon dioxide (PaCO_2_) and pH, and FiO_2_ and PEEP to maintain pulse arterial oxygen saturation (SpO_2_) between 90 and 95% (25, 26).

#### 2.4.2 Transition of control modes to PSV

During the morning rounds, the responsible ICU physicians check the ventilator mode for each patient. The ventilator mode is recommended to transit from controlled modes to PSV if all the following criteria are met:

1. The patient is able to trigger ventilator breaths;
2. PaO_2_/FiO_2_ above 100 mmHg;
3. PEEP below 15 cmH_2_O;
4. Stable hemodynamic status (none or stable doses of vasopressors);
5. No sedation or light sedation ([RASS] of −2 to +1 or [SAS] of 3 to 4).

#### 2.4.3 PSV setting strategy

In both the PMI-targeted and VT/RR-targeted groups, the same principle PSV settings are followed except for the adjustment of pressure support level:

1. The inspiratory trigger sensitivity is set as 1–2 L/min for the flow-trigger or 1.5–3 cmH_2_O for the pressure-trigger;
2. Inspiration-to-expiration cycle-off is set as 25% of the peak inspiratory flow. Responsible physicians can adjust this parameter in case of suspected cycle-off patient-ventilator asynchrony;
3. FiO_2_ and PEEP are set to maintain SpO_2_ between 90 and 95%. Specifically, if SpO_2_ is lower than 90%, PEEP and then FiO_2_ will be increased by 2 cmH_2_O and 0.1, respectively; whereas if SpO_2_ is higher than 95%, FiO_2_ and then PEEP will be decreased by 0.1 and 2 cmH_2_O, respectively.

**Figure 2:**
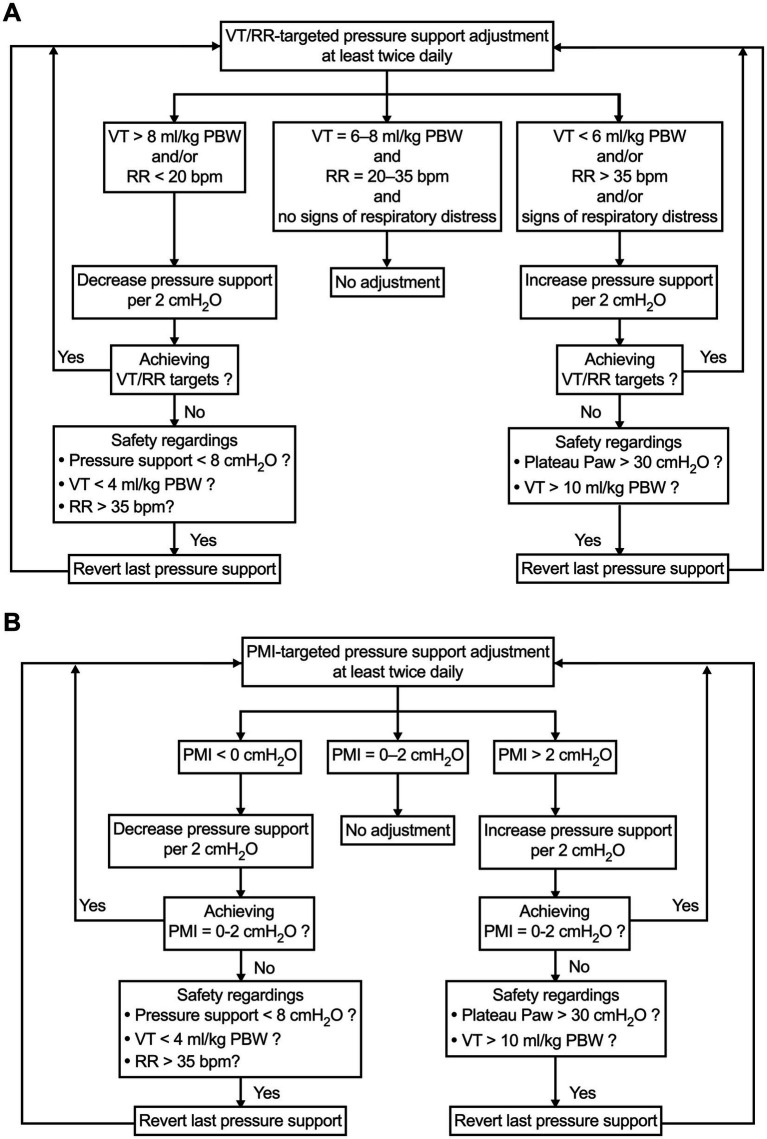
Pressure support setting and adjustment strategy in VT/RR-targeted group **(A)** and PMI-targeted group **(B)**. PBW, predicted body weight; PMI, pressure muscle index; RR, respiratory rate; VT, tidal volume.

In the VT/RR-targeted group, the pressure support is adjusted to obtain a VT between 6 and 8 mL/kg PBW, RR between 20 and 35 breaths/min, and no signs of respiratory distress, such as prominent use of the accessory respiratory muscles, nose flaring, retractions, etc. (Figure 2A).

In the PMI-targeted group, the pressure support is adjusted according to the algorithm shown in Figure 2B. During PSV, after an end-inspiratory airway occlusion, Paw will reach a plateau. PMI can be measured on the ventilator screen as plateau Paw minus peak Paw by using a sliding marking line when the screen frozen function is initiated. According to our previous studies, a PMI value of 0 to 2 cmH_2_O will be used as the target for pressure support adjustment. This PMI range represents a well-accepted normal inspiratory effort (19, 20).

During the study period in the two groups, pressure support adjustment will be performed at least twice daily.

#### 2.4.4 Rescue backup of controlled ventilation

In both groups, PSV can be switched to protective controlled modes when predefined criteria are met, which include at least one of the following conditions:

1. Pressure support level above 20 cmH_2_O;
2. PEEP above 15 cmH_2_O;
3. pH < 7.30;
4. PaO_2_/FiO_2_ ≤ 100 mmHg;
5. Strong inspiratory effort which cannot be controlled by sedation;
6. Unstable hemodynamic status (systolic blood pressure below 90 mmHg with vasopressors or systolic blood pressure above 180 mmHg);
7. Active cardiac ischemia;
8. Unconsciousness (RASS < −3 or SAS < 2);
9. Dangerous agitation that cannot be controlled by sedation (RASS > +2 or SAS > 6).

Data will be documented in patients whose mechanical ventilation is switched to controlled modes. The patients will be reassessed at least every 12 h and transited back to PSV as soon as possible according to the abovementioned criteria. The patients will be visited daily until 28 days after randomization. Data will be documented and reported for those patients whose controlled ventilation is not transited back to PSV during the follow-up.

#### 2.4.5 Weaning and extubation

The weaning and extubation process follows clinical guidelines recommended by the American Thoracic Society and the American College of Chest Physicians (27).

The attending physicians assess readiness to initiate the weaning process during daily morning rounds. If all the following aspects are satisfied, an SBT will be performed:

1. Improvement of the cause of intubation and mechanical ventilation;
2. Hemodynamic stability when the dose of norepinephrine is less than 0.1 μg/kg/min or equivalent and lasts for 6 h at stable or reduced doses;
3. No agitation or coma;
4. SpO_2_ higher than 90% with FiO_2_ less than 0.4 and PEEP less than 5 cmH_2_O.

SBT is conducted by respiratory therapists using the low-level PSV with pressure support of 5 cmH_2_O and PEEP of 5 cmH_2_O. The SBT will last at least 30 min, and criteria for failure of SBT include:

1. RR of greater than 35 breaths/min for more than 5 min;
2. SpO_2_ of less than 90%;
3. Elevated PaCO_2_ and/or pH of less than 7.3;
4. Heart rate (HR) of greater than 140 beats/min or a sustained change in HR of more than 20%;
5. Systolic blood pressure greater than 180 mmHg or less than 90 mmHg;
6. Signs of cardiac ischemia indicated by dynamic ST changes on ICU monitor or electrocardiogram;
7. Abrupt unconsciousness with RASS below −3 or SAS below 2;
8. Signs of anxiety, agitation, or diaphoresis.

Patients with a failure SBT will be mechanically ventilated with PSV following settings and adjustments according to the original grouping.

Extubation will be performed in endotracheal intubated patients who pass the SBT, and mechanical ventilation will be discontinued in patients with tracheostomy. PSV will be restored to maintain the grouping in patients with reintubation or reapplication of mechanical ventilation via tracheostomy within 7 days. The decision to reintubate and restore mechanical ventilation will be at the discretion of the ICU physician team according to the local standard of care. The duration of mechanical ventilation will be added to the total duration.

### 2.5 Data collection

An electronic case report form (eCRF) is designed and available online via the SoJump platform (Changsha Ranxing Information Technology Co., Ltd, Hunan, China) (online table). Before the start of the study, clinical research coordinators who have received training in data collection are assigned to each participating ICU.

Data will be anonymous and coded with a three-level structure (Figure 1B). Level 1 represents each enrolled patient, who will be assigned a unique three-digit number. In Level 2, each cluster (ICU) will be assigned a unique two-digit number. In level 3, clusters randomized to the PMI-targeted or the VT/RR-targeted group will be assigned number one or two, respectively. The eCRF will automatically assign a data code to each patient as xxx-xx-x (patient-cluster-group).

#### 2.5.1 Data collected at the study entry

At baseline, demographics, comorbidities, diagnosis for ICU admission, recent medical history, reasons for mechanical ventilation, duration of ventilation before enrolment, conditions of gas exchange, and mechanical ventilation settings will be documented, as will the Acute Physiological and Chronic Health Evaluation II (APACHE II) score at the ICU admission.

#### 2.5.2 Data collection during daily visits

All patients will be visited daily between 08:00 and 12:00 until successful weaning or separation of mechanical ventilation, death, hospital discharge, or until 28 days after randomization. Successful weaning or separation of mechanical ventilation is defined as extubation without reintubation or death within the next 7 days, whether post-extubation noninvasive ventilation is used or not, or ICU discharge without invasive mechanical ventilation within 7 days, whichever comes first (28).

Data collected include:

1. Gas exchange: SpO_2_ and arterial blood gas analysis;
2. Hemodynamics: blood pressure, HR, vasoactive agents, and cumulative fluid balance during the last day;
3. Analgesia and sedation: RASS or SAS score, visual analog scale or critical care pain observation tool, the use of analgesics and sedatives;
4. Sequential Organ Failure Assessment (SOFA) score;
5. Switch to controlled ventilation: whether switching to controlled mode during the last 24 h, reasons, and settings;
6. PSV: pressure support level, PEEP, FiO_2_, VT, and RR;
7. Respiratory drive: the negative airway pressure generated during the first 100 ms against an end-expiratory airway occlusion (airway occlusion pressure, P0.1) (29);
8. SBT: whether performing an SBT during the last 24 h, methods, and results;
9. Extubation or discontinuation of mechanical ventilation: whether performed during the last 24 h;
10. Re-intubation or restoration of mechanical ventilation: whether performed during the last 24 h and reasons;
11. Tracheostomy: whether performed during the last 24 h;
12. Self-extubation: whether occurring during the last 24 h;
13. 28-day follow-up: duration of mechanical ventilation, length of stay in the ICU and hospital, and death.

### 2.6 Outcome measures

#### 2.6.1 Primary outcome

The primary outcome is the ventilator-free days (VFDs) at day 28 after enrollment. The calculation of VFDs will follow the standard recommendations (30, 31).

#### 2.6.2 Secondary outcomes

Secondary outcomes include:

1. Duration of mechanical ventilation before enrollment;
2. Total duration of mechanical ventilation;
3. The time before the first SBT, which is defined as the time interval from intubation and mechanical ventilation to the first SBT attempt;
4. Weaning time, which is defined as the time from the first SBT attempt to successful discontinuation of mechanical ventilation (28);
5. Frequency of prolonged weaning, which is defined following WIND classification (28);
6. Frequency of self-extubation, re-intubation, tracheotomy, and mechanical ventilation longer than 21 days
7. Length of stay in the ICU and hospital;
8. ICU mortality, hospital mortality, and 28-day mortality.

### 2.7 Training and quality control

After randomization, a four-week comprehensive training program will be conducted for all staff in the participating ICUs before the formal start of the trial.

The program includes:

1. Two 30 min online hands-on training “PSV initiation, pressure support adjustment, and weaning process” courses, each for the PMI-targeted and VT/RR-targeted group, will be separately held for staff in ICUs according to the grouping;
2. The videos of these courses will be disseminated to the participating ICUs, and the local primary investigators will be responsible for organizing local training;
3. A formal training and operational manual will be composed and disseminated to the participating ICUs;
4. Chief investigators (FXZ, LW, and JXZ) will outreach visit each participating ICU to provide on-site hands-on training before the start of the trial.

We will pay special attention to measuring PMI in the training program for the PMI-targeted group. According to our previous study (20) and recommendations provided by Bianchi et al. (32), the training course demonstrates a standard performance of end-inspiratory airway occlusion. Physicians, nurses, and respiratory therapists in the PMI-targeted group will be instructed and trained to perform air leak checks, to observe the flow-time waveform during occlusion, and to obtain a longer than a 2 s duration of plateau Paw.

During the study, some active and passive measures are conducted for quality control:

1. One clinical research coordinator will be assigned to each participating ICU;
2. Monthly site inspections and audits of data collection will be carried out at each ICU by the central coordination group;
3. A pocket card with the instructions for trial intervention is designed and will be disseminated to the ICU staff;
4. A color poster is designed and will be placed on the head of each enrolled patient’s bed.

### 2.8 Safety

A safety monitoring board composed of three independent experts on mechanical ventilation who do not participate in the conduct of the trial will supervise the safety of the study.

On the basis of clinical experience and the results of physiologic studies (18–20), the additional risks for patients enrolled in the PMI-targeted group are expected to be minimal in comparison to the standard of care employed in the VT/RR-targeted group. Nonetheless, patient insurance will be granted to cover all unexpected adverse events caused by the trial interventions. All adverse events will be monitored and reported to the safety monitoring board. The board will review and examine the report and provide written recommendations to the chief investigators.

### 2.9 Sample size and statistical analysis

#### 2.9.1 Sample size estimation

Our previous studies showed that the mean (± standard deviation) VFDs in Chinese academic hospitals was 19 ± 3.3 days (19, 20). An individual RCT will require 342 patients to detect a one-day increase in VFDs with a type I error (Alpha) of 0.05 and a power of 80%. According to previous studies, an intra-cluster correlation coefficient of 0.03 is used to calculate the sample size for the cluster RCT (33, 34), which results in a target of 551 recruited patients. Accounting for 15% of immeasurable PMI during PSV (19, 20), a sample of 648 will be the target, with an approximate average recruiting 40 patients in 16 participating ICUs.

#### 2.9.2 Statistical analysis plan

All statistical analyses will be conducted in accordance with the intention-to-treat principle. That is, all randomized patients will be analyzed in the groups to which they were originally allocated. Researchers performing statistical analysis will be blinded to the cluster grouping.

Categorical variables will be presented as numbers and percentages and compared using the chi-squared test or Fisher’s exact test. For continuous variables, normal distribution will be checked by Kolmogorov–Smirnov Test, and presented as mean and standard deviation or median and interquartile range, as appropriate. Comparison of continuous variables will be performed by Student’s *t*-test for normally distributed variables and the Mann–Whitney U test for non-normally distributed variables.

Time-to-event variables will be analyzed using survival analysis, and the difference between groups will be assessed with the log-rank test.

The patients will be stratified *a priori* into subgroups with:

1. high versus intermediate and low respiratory drive according to the P0.1 criteria reported by a previous study (29);
2. diagnosis of acute respiratory distress syndrome (ARDS) versus non-ARDS according to the Global definition (35);
3. long versus short duration of controlled ventilation before enrollment according to the median obtained in all enrolled patients.

Statistical analyses will be performed using MedCalc (2022 MedCalc Software Ltd., Belgium). A *p* value of less than 0.05 is considered statistically significant.

## 3 Discussion

In the current clinical practice of PSV, a combination of VT and RR is recommended as the target inspiratory support level (7, 8), and many centers follow this strategy (36–38). However, previous studies have shown that excessive support of inspiratory pressure may not be uncommon based on this approach (13, 14). Additionally, a mismatch between patient demand and ventilatory support is the main reason for patient-ventilator asynchrony, which may make weaning difficult and lead to prolonged mechanical ventilation (39, 40). Therefore, monitoring the patient’s inspiratory effort and tailoring the pressure support to match the effort is a reasonable solution.

Previous studies have suggested that the PMI, which can conveniently be obtained on the ventilator screen, could reliably detect low and high inspiratory effort (18–20). In patients undergoing PSV, our study group also found the range of PMI to predict the high and low contribution of a patient’s effort and the feasibility of pressure support adjustment according to the PMI target (20).

In this cluster RCT, the major intervention is comparing the pressure support setting and adjustment strategy between the VT/RR target (traditional strategy) and the PMI target (modified strategy). Randomization within one ICU may result in confusion among staff caring for the patients and a high likelihood of crossover during the study. Therefore, we chose a cluster RCT design rather than an individual RCT to avoid contamination.

The design of the study procedures is based on two main principles: rigorousness and feasibility. In the study protocol, we predefine standard clinical management of PSV, including the initiation of PSV, PSV setting strategy depending on the grouping, rescue backup of controlled ventilation modes, and weaning and extubation process. These components of the procedures are in accordance with the international guidelines (25–28) and followed by the participating ICUs. Additionally, we reinforce these procedures through comprehensive training before the initiation of the trial and the use of active and passive measures of quality control during the study. On the other hand, for the feasibility of the study, we mainly focus on the application of pressure support setting and adjustment strategy with a limited burden on the intervention workload.

A clear algorithm for pressure support adjustment is designed in either the VT/RR-targeted or PMI-targeted group (Figure 2). In both groups, safety considerations are schemed in case the adjusted pressure support level does not achieve the target. We anticipate that these algorithms will be highly operable in the study’s performance.

Post-hoc analysis will be performed by predefine stratification into a subgroup with high inspiratory effort, a subgroup with ARDS, and a subgroup with a long duration of controlled ventilation before enrollment. Such subgroup analysis may help identify patient populations that may benefit from pressure support adjustment strategies based on effort assessment.

There are two major potential problems in the present study. The first is the accessibility of a stable plateau Paw during end-inspiratory occlusion and the measurement of PMI. This maneuver is not widely applied in clinical practice because airway occlusion may induce an unstable plateau Paw (32). However, relatively high incidences of unstable occluded Paw were reported in retrospective PMI analyses in which the main purpose was not the measurement of plateau pressure in the original studies (16, 41). In our previous study, we integrated a training and quality control approach emphasizing several key points during the end-inspiratory occlusion (check of air leak, observation of zero flow during the occlusion, and a length of occlusion longer than 2 s) (19, 20). Incidence of immeasurable plateau pressure decreased to less than 10%. These results were confirmed by a recently published study by another research group (18). Second, in our experience and results from published investigations (42), it seems that PSV is only used as a mode for preparing the liberation of mechanical ventilation in many Chinese ICUs. However, an international prospective cohort study demonstrated that the use of PSV had increased over the past two decades and became the most commonly used assisted ventilation mode during the acute phase of critical illness (2). As mentioned above, the training in standard clinical management of mechanical ventilation, especially for the initiation of PSV, will be emphasized before the start of the trial. We hope this approach might enrich our recruitment. However, because there is substantial heterogeneity in the mechanically ventilated patients, subtypes of patients may have a different response to the support pressure adjustment. For example, patients with AHRF are quite different from those with ARDS (43). Although we predefined post-hoc subgroup analysis, future work with prespecified subgroups will be needed and helpful, such as patients with abnormal low or high inspiratory drive and effort.

In conclusion, IT-PSV is a cluster RCT that will evaluate the effect of a PMI-targeted pressure support setting and adjustment strategy on the duration of mechanical ventilation in patients undergoing PSV. If positive, it will provide a new physiological-based PSV management that could potentially facilitate protective assisted ventilation.

### Dissemination policy

The data generated in the present study will be available from the corresponding author upon reasonable request. The results of the trial will be submitted to press conferences and/or international peer-reviewed journals.
